# Supplementary material for: Min-max approach for comparison of univariate normality tests
Source: PLoS One. 2021 Aug 4;16(8):e0255024. doi: 10.1371/journal.pone.0255024 (PMC8336887; doi:10.1371/journal.pone.0255024)
Supplement: Attachment — Submitted filename: Response to Reviewers.docx [file pone.0255024.s001.docx]

Saeed Mian Qaisar, Ph.D.

Academic Editor

PLOS ONE

Manuscript ID: PONE-D-21-14331

Dear Dr. Saeed Mian Qaisar,

Thank you for inviting me to submit a revised draft of our manuscript entitled, "Min-Max Approach for Comparison of Univariate Normality Tests" to PLOS ONE. I also appreciate the time and effort you and each of the reviewers have dedicated to providing insightful feedback on ways to strengthen my paper. Thus, it is with great pleasure that I resubmit my article for further consideration. I have incorporated changes that reflect the detailed suggestions you have graciously provided. I also hope that my edits and the responses I provide below satisfactorily address all the issues and concerns you and the reviewers have noted.

To facilitate your review of my revisions, the following is a point-by-point response to the questions and comments delivered in your letter dated June 16 2021.

**Journal Requirements:**

1. Please ensure that your manuscript meets PLOS ONE's style requirements, including those for file naming.

**Response:** I have followed all the style requirements including the file names.

2. Upon re-submitting your revised manuscript, please upload your study’s minimal underlying data set as either Supporting Information files or to a stable, public repository and include the relevant URLs, DOIs, or accession numbers within your revised cover letter.

**Response:** This is a simulation based study and all the codes required to generate data or replicate the study are deposited on the Github as suggested by one of the reviewer. The URL of the file is included in the revised cover letter.

**Reviewer # 1 Comments:**

I appreciate the time and energy the anonymous reviewer committed and provided me the valuable feedback for the improvement of the manuscript.

1. In section 2, distribution of the RJB test should be mentioned as well.

**Response:** The distribution is mentioned on pg#7, line# 132-33.

2. In table 3 & 5, how the ranks are defined for tests with small differences in losses?

**Response:** The ranking changes if the difference in losses is more than 2%.

3. Fig. 1 & 2 highlight the deviations of JB and RJB test against the worst alternatives at sample of sizes 25 and 75. Do the tests behave alike at sample of size 50? If yes, add this information as footnote or in the main text.

**Response:** Thanks for highlighting the missing information. Yes, the tests behave alike at sample of size 50 as well and this information is added in main text at pg#21, line# 335-36.

4. Table numbers are not aligned, e.g., there are two tables titled as table 5.

**Response:** This typo is corrected on pg# 25, line# 397.

5. It is evident from literature that the JB and RJB tests behave well against long-tailed alternatives. This study should acknowledge the same (as it is evident from this study results as well) and particularly mention that there are only few long-tailed worst alternatives which should be taken care of while testing normality assumption by applying the JB and RJB tests.

**Response:** Thank for the suggestion! I have acknowledged the same on pg# 32, line# 463-64.

**Reviewer # 2 Comments:**

I am thankful to you for providing me the valuable comments for the value addition to my work.

1. More details on the simulations should be disclosed. For example, which software do you use? Additionally, in order to facilitate the reproduction of the study you should provide data. If you generate your distributions with Python, R, Matlab, etc, you could include the code as a supplementary material or upload at a repository such as Github.

**Response:** Thanks for providing guidance regarding the repository like Github. I have uploaded the codes required to generate the data for simulations and code for the replication of the study as well. The URL of the file is included in the revised cover letter. The software information is added to the manuscript at pg# 16, line# 275.

2. Probably you could complement your results using a classification or a clustering algorithm. In other words, which features of the distribution influences the loss function the most? Do some tests perform better at detecting non normality against a Beta rather than against a Laplace distribution? You could use Self Organizing Maps or K-means or many other techniques in order to produce such representation. In this way instead of a ranking such as in tables 5,6,7, you could produce a ranking that informs on the different dimensions that affect the normality detection.

**Response:** Thanks again for the valuable comment, it helped me to improve my analysis of results. I have highlighted the top-five damaging distributions for each selected normality test at all sample sizes in table 8. This would help researchers to choose the best normality test based on the data distribution in hand.

Again, thank you for giving me the opportunity to strengthen my manuscript with your valuable comments and queries. I have worked hard to incorporate your feedback and hope that these revisions persuade you to accept my submission.

Your Sincerely,

Tanweer Ul Islam,

National University of Sciences & Technology Islamabad, Pakistan, 44000.

Email: [tanweer@s3h.nust.edu.pk](mailto:tanweer@s3h.nust.edu.pk) ; Tel: +92-51-9085 3567
